# Recent developments in treatment of latent tuberculosis infection

**Published:** 2011-03

**Authors:** Dick Menzies, Hamdan Al Jahdali, Badriah Al Otaibi

**Affiliations:** *Montreal Chest Institute, McGill University, Montreal, Quebec, Canada*

**Keywords:** Latent tuberculosis, tuberculosis, tuberculosis infection, tuberculosis prevention, tuberculosis treatment

## Abstract

Latent tuberculosis infection (LTBI) can be detected with immune based tests such as the tuberculin skin test (TST) or interferon gamma release assays (IGRA). Therapy for those with positive tests can reduce the subsequent risk of re-activation and development of active TB. Current standard therapy is isoniazid (INH) which reduce the risk of active TB by as much as 90 per cent if taken daily for 9 months. However, this lengthy duration of therapy discourages patients, and the risk of serious adverse events such as hepatotoxicity, discourages both patients and providers. As a result completion of INH therapy is less than 50 per cent in many programmes. However, programmes that offer close follow up with supportive staff who emphasize patient education, have reported much better results. The problems with INH have stimulated development and evaluation of several shorter regimens. One alternative was two months daily rifampin and pyrazinamide; this regimen has been largely abandoned due to unacceptably high rates of hepatotoxicity and poor tolerability. The combination of INH and rifampin, taken for 3 or 4 months, has efficacy equivalent to 6 months INH albeit with somewhat increased hepatotoxicity. Four months rifampin has efficacy at least equivalent to 6 months INH but there are inadequate trial data on efficacy. The safety of this regimen has been demonstrated repeatedly. Most recently, a regimen of 3 months INH rifapentine taken once weekly under direct observation has been evaluated in a large scale trial. Results have not yet been published, but if this regimen is as effective as INH, this may be a very good alternative. However, close monitoring and surveillance is strongly suggested for the first few years after its introduction. Evidence from several randomized trials has shown that the benefits of LTBI therapy is only in individuals who are tuberculin skin test (TST) positive even among those with HIV infection. Hence, LTBI therapy should be given only to those with positive tests for LTBI. We conclude that LTBI therapy is considerably underutilized in many settings, particularly in low and middle income countries.

## Introduction

It has been estimated by the World Health Organization (WHO) that more than one third of the entire world’s population, or approximately 2 billion persons are infected and can be considered carriers of TB. From this vast pool of persons with latent TB infection (LTBI) approximately 200 million will develop active TB during their lifetime^1^. More than 80 per cent of these will be contagious and transmit TB infection to others, perpetuating the cycle of TB and resulting in continued morbidity and mortality from TB for generations to come^2^. The impact of TB is even greater than these numbers may suggest, as 75 per cent of individuals with TB are within the economically productive age group of 15-54 yr^2^, and TB disproportionately affects low and middle income countries, as 95 per cent of all cases and 99 per cent of all deaths occur in these countries^2^.

Two approaches can be taken to reduce the burden of TB over the long term. One approach requires early and rapid diagnosis of all persons with active TB with institution of effective therapy to render them non contagious. This will reduce transmission, reducing the number of carriers, who will later develop active TB. However, WHO has estimated that less than 50 per cent of all cases globally are currently diagnosed and treated^1^. Since each person with undiagnosed and untreated active TB will survive an average of 2 yr, it has been estimated that they will infect 20 others^2^. An additional problem is that most patients in high incidence settings are diagnosed by direct smear microscopy of unconcentrated sputum specimens^3^, which only detects patients with advanced disease. Hence, significant transmission can occur prior to diagnosis^2^.

An alternate approach is to detect persons while they still have LTBI and treat them to prevent the later development of disease. This approach dates back to the 1950s. Soon after isoniazid (INH) was introduced as an effective agent to treat disease, clinicians noted that INH monotherapy effectively prevented development of disease in children with primary TB^4^. Following this clinical observation, more than 20 randomized trials, involving more than 100,000 participants, were conducted in more than a dozen countries^5^. These studies demonstrated that INH taken for at least 6 months in persons with LTBI reduced subsequent TB incidence by 25 to 92 per cent. The differences in effectiveness found in different studies were largely explained by differences in treatment completion^5^.

However, INH treatment of LTBI is problematic for several reasons. The lengthy duration of treatment reduces patient compliance^6^–^8^, while the potential occurrence of serious adverse events such as hepatitis, further discourages patients’ and providers’ acceptance of this therapy^9^,^10^. As a result there has been considerable interest and many evaluations of alternative shorter regimens. This work continues even today.

The objectives of the present article are to *(i)* review the evidence from systematic reviews and recent trials of efficacy, completion, and adverse effects of several commonly used regimens for treatment of LTBI; *(ii)* review risk-benefit and other considerations for treatment of LTBI in high income, as well as low and middle income countries; and *(iii)* review the indications for tuberculin testing (or use of other methods of diagnosis of LTBI) prior to starting LTBI treatment.

## Regimens for treatment of LTBI

## 6 Months INH (6INH)

Numerous trials have evaluated different durations of INH^11^. However, only one clinical trial has directly compared regimens of different durations of INH. The International Unit Against Tuberculosis (IUAT) trial, conducted in Eastern Europe, randomized approximately 28,000 individuals with positive tuberculin skin tests (TST) and fibronodular changes on chest X-ray^12^. Approximately 7,000 participants each were randomized to placebo, 3, 6 or 12 months of INH (Table I). Compliance was assessed with monthly pill counts. Compared to participants who took placebo, participants who completed 3 months INH had 31 per cent reduction in TB; this effect waned over the 5 years of follow up. Participants who completed 6 months INH had 69 per cent reduction of active TB, while subjects who completed 12 months INH (12INH) had 93 per cent reduction in TB. The efficacy of 6INH and 12INH waned during the 5 yr of followup but always remained significantly better than placebo^12^. However, completion of the 12 month regimen was much less than the 6 month regimen.

**Table I:** Summary of LTBI regimens in common use

Regimen	Efficacy (relative to placebo)#	Completion of therapy	Serious adverse events	
			Type	Rate

INH: 6 months	69%^12^	50%^6^,^8^,^54^	Hepatitis	1 -5%^23^,^68^
INH: 9-12 months	90 – 93%^12^,^17^	<50%^31^,^33^,^34^,^69^	Hepatitis	1 -5%^23^,^31^,^34^,^68^
RIF&PZA: 2 months	Equal to 6 -12INH^27^,^63^	6% > 6-12INH*^27^	Hepatitis	3 -5%^27^,^68^,^70^
INH&RIF: 3-4 months	Equal to 6INH^30^,^36^	6% > 6-12INH**^36^	Hepatitis†	1 – 5%^36^,^37^
RIF: 3-4 months	65% ##^30^	22% > 9INH***^31^–^34^,^71^	Rash Hepatitis	1- 2%^31^,^34^
				<1%^31^,^32^,^34^

Superscript numerals denote reference nos.; INH, isoniazid, 6 INH, 6 months isoniazid; 6-12 INH, 6-12 months INH; PZA, Pyrazinamide; RIF, Rifampins;

# Efficacy from placebo randomized trials. If trials were not placebo controlled then efficacy relative to INH is given;

## Based on one placebo-controlled trial with 3 months rifampin (30);

* Average 6% (range: -5 to 19%) better completion than with 6-12INH;

** Average 6% (range: 3 to 12%) better completion than with 6–12INH;

*** Average 22% (range: 18 to 27%) better completion than with 9INH;

† Hepatitis more frequent with INH&RIF than INH alone (37); Figures in parentheses denote Ref numbers

Based on these results, and assuming that under programme conditions completion of longer regimens would be equally poor, if not worse, many authoritative agencies have recommended 6INH as an acceptable alternative to 12INH^13^,^14^. Snider and colleagues^15^ estimated that 6INH would be more cost-effective than 12INH. This reflects the fact that the majority of costs of LTBI therapy is related to the initial screening, subsequent medical evaluation and start of therapy^16^. However, it is important to remember that in patients who take therapy properly, 12INH is significantly and substantially more efficacious than 6INH.

## 9 Months INH (9INH)

Concerns regarding the relatively low efficacy of 6INH, and equally serious concerns regarding the poor completion of 12INH resulted in recommendations for 9 months INH by the American Thoracic Society in 2000^13^. This recommendation was based almost entirely upon a re-analysis by Dr George Comstock of data from several INH trials among Alaskan Inuit populations^17^. This re-analysis demonstrated that the optimal duration of INH was 9 months, with estimated efficacy of 90 per cent and no significant gain with extension to 12 months. Hence, 9INH was recommended as the standard of care by the American Thoracic Society in 2000^13^, and other authorities followed suit soon after^14^.

Completion of 9INH under programme conditions is poor. Many programmes report less than 50 per cent completion although a few programmes report much higher completion rates^18^,^19^. These programmes emphasize initial intensive patient education, with subsequent close follow up, and patient support; they demonstrate that many patients can be motivated to complete 9 months therapy.

However, INH has the major disadvantage of potential serious adverse events. Of particular concern is hepatotoxicity, as this is difficult to detect, and can be fatal. Interestingly in the randomized trials conducted up to 1970 this problem had not been noted^20^. Hence when INH was recommended by the American Thoracic Society (ATS) in 1971, the potential for adverse effects was considered minimal, and this treatment was strongly recommended for TST reactors of all ages^21^. With the ensuing much greater use, serious hepatotoxicity causing deaths was described^22^. Subsequent surveillance studies confirmed that hepatotoxicity was quite common in patients taking INH, and could be severe, with up to 1 per cent mortality in older patients^23^. Subsequently INH related mortality has declined over the last 30 yr^24^,^25^ – presumably related to better selection of patients for therapy and closer follow up.

## 2 Months rifampin –pyrazinamide (2RZ)

Studies of latent TB infection in a mouse model revealed that 2 months of treatment with a combination of rifampin and pyrazinamide (PZA) resulted in complete clearance of TB bacilli - a bacteriologic effect comparable to that achieved with 6 months INH in the same mouse model. Following publication of these results in 1989^26^, several randomized trials were conducted to compare 6 to 12 months INH with 2RZ in HIV infected patients^27^. These trials demonstrated equivalent efficacy; however, since most compared 2RZ to 6INH this would indicate that efficacy of 2RZ was likely less than the ideal achieved with 9INH. Serious adverse events were not more frequent with 2RZ than with INH in these trials. However, completion of 2RZ was not significantly better than completion of 6INH in several of these trials, despite the shorter duration^27^. In retrospect, this may have been a harbinger of events to come.

In 2000, based on these promising trial results, the American Thoracic Society recommended use of 2RZ^13^. This was a strong recommendation for use in HIV infected persons, and a conditional (or weak) recommendation for non-HIV infected persons. Publication of these recommendations was quickly followed by rapid, enthusiastic and widespread use of 2RZ. In turn this was quickly followed by reports of serious hepatotoxicity and death^28^. Subsequent trials in non HIV infected individuals revealed very high rates of grade 3 to 4 hepatotoxicity, which sometimes occurred at the end or even after completion of therapy^27^. As a result recommendations were revised that 2RZ should be used with caution in HIV infected and extreme caution in non HIV infected^29^.

Our current practice is to consider use of this regimen in HIV infected individuals in situations where close supervision and monitoring is possible and longer therapy LTBI regimens may be impractical. We do not recommend this regimen in non-HIV infected persons.

## 4 Months rifampin (4RIF)

An overlooked finding in the same study of treatment of latent LTBI in mice was that rifampin alone for 2 months was equally efficacious as 2RZ, or 2 months of rifampin plus- INH^26^. Complete bacteriologic clearing had occurred in all TB infected mice by the end of two months mono-RIF therapy. Unfortunately, despite this very promising evidence, only one randomized trial has evaluated a regimen of rifampin alone. In this trial, older men living in Hong Kong, who had pulmonary silicosis with a positive TST were randomized to placebo, 6INH, 3 months INH plus Rifampin, or 3 months Rifampin alone^30^. During five years of follow up, 27 per cent of those randomized to placebo developed active TB, compared to 16, 13, and 10 per cent for the three regimens respectively. The estimated effectiveness of 3 months rifampin was approximately 65 per cent; this was better than the other regimens although the differences between active regimens was not significant, and all were significantly better than placebo^30^. To date, there are no other published trial evaluating mono-RIF therapy. However, a large scale trial comparing the effectiveness and efficacy of 4RIF and 9INH is ongoing.

Several studies have reported that completion rates with 4RIF are substantially and significantly better than with 9INH^31^–^34^. In addition, rates of serious adverse events with 4RIF are very low, particularly rates of hepatotoxicity^31^. In a recently published randomized trial rates of grade 3 to 4 adverse events were significantly lower with 4RIF than 9INH. Grade 3-4 hepatotoxicity occurred in 4 per cent of patients taking 9INH compared to less than 1 per cent in those taking 4RIF^34^. The earlier results in the Hong Kong silicosis trial suggest that 4RIF should have efficacy of at least 65 per cent^30^- equivalent to the documented efficacy of 6INH.

In summary, 4RIF appears to be well tolerated, has excellent completion and a very good safety record. However, 4RIF cannot be recommended for routine use until the results of the efficacy trial are available.

## 3 to 4 Months of INH and RIF (3-4INH-RIF)

The animal study described earlier suggested that INH and RIF for 2 months was effective in the mouse model. Uncontrolled trials in paediatric populations have suggested that this regimen should be adequate^35^. A series of randomized trials^36^, have demonstrated that the efficacy of 3-4INH-RIF is equivalent to that of 6INH (4 studies) or 9INH (1 study) although adverse events are significantly more frequent^37^. This latter finding is not that surprising, since the majority of hepatotoxic adverse events with INH occur in the first 2-3 months^34^,^38^. Therefore adding INH to rifampin for 3 or 4 months will inevitably increase the risk of adverse events. However, the benefits of adding the INH to RIF are unclear. In theory, therapy with two agents will provide a margin of safety if the person has minimal active TB rather than true latent TB infection. There is no evidence of improved efficacy compared to RIF alone, and to justify LTBI therapy on the basis of potential misdiagnosis of active TB as latent TB seems a fairly weak rationale for this regimen.

## New regimens

A trial comparing 9INH with 3 months of once weekly directly observed INH combined with rifapentine (3INH-RPT) has just been completed. Rifapentine is a rifamycin with half life that is 5 times longer than rifampin - allowing once weekly therapy. More than 8,000 participants were randomized. Completion of 3INH-RPT was 84 per cent, compared to 71 per cent completing 9INH. Efficacy and safety data are still being analyzed, but results are expected soon. If these are acceptable, this may be an excellent alternative regimen. However, the requirements for directly observed preventive therapy may make this impractical in some settings.

In addition, the experience with INH in 1970, and the 2RZ regimen in 2000 should provide a cautionary note for implementation of this new regimen. For both of these regimens the potential for serious and even fatal adverse events was not detected in multiple randomized trials. The serious hepatotoxicity of both regimens was only recognized after these had been recommended and implemented widely in routine practice. Rifapentine is a relatively new and untested drug. Hence this drug may cause unexpected adverse events that were not detected in the randomized trial. Given the long half life of this drug the adverse events may progress even after treatment is stopped, resulting in more serious sequelae. Hence, introduction of this new regimen should be accompanied by careful monitoring and evaluation of adverse events.

## Drug-resistant LTBI

Treatment of close contacts of drug resistant active cases is difficult and yet is an increasingly common clinical problem. For contacts of INH resistant index cases, INH will be ineffective^39^, so 4RIF is recommended^13^,^14^. Contacts of index cases with MDR TB are recommended to receive 6 months of PZA and a quinolone^13^. However, two observational studies have reported very high rates of toxicity and extremely poor completion due to intolerance^40^,^41^. In both series no patients actually completed the proposed 6 month course of therapy. Based on this and our own clinical experience which was similar, our current practice is to give moxifloxacin or levofloxacin alone for 6 months. The rationale for this monotherapy is the published evidence that moxifloxacin can replace INH in treatment of active TB^42^.

## Consideration for use in different settings

Over the past 30-40 yr treatment of LTBI has been a major priority for TB control programmes in many high income countries such as Canada, U.S.A., Australia or Saudi Arabia. However, LTBI diagnosis and treatment has been a low priority for TB control programmes in most low and middle income countries (LMIC). This reflects the cost and resources required for effective and safe treatment of LTBI, given the need for close follow up and monitoring to detect serious adverse events and to encourage and monitor compliance with therapy. Until an LTBI regimen is found that is perfectly safe and very easy to take, these resource requirements will be present. Medication costs are low, but micro costing studies have shown that medications account for less than 10 per cent of total resources used^16^,^43^. Nevertheless therapy of individuals with LTBI and important risk factors for reactivation such as HIV co-infection, or close contacts is warranted in all settings as this therapy provides significant individual benefits, and is much more cost-effective.

In high income settings, the costs are not as determinant of LTBI therapy as risk-benefit considerations. After realization that serious adverse events and even deaths could occur with INH, several investigators performed decision analyses to estimate the risks and benefits of INH therapy. As shown in Table II, the majority of these studies examined the risks and benefits of treatment with INH in healthy individuals with a positive tuberculin skin test but no other risk factors. In these individuals the benefits of INH therapy are low because the life-time risk of development of active TB is low. Therefore, the benefits barely exceeded the risks. And, because of slightly different assumptions made by the different investigators, INH therapy was found to result in small net gains or losses of life expectancy. However, the absolute differences in findings between these studies were very small^44^–^48^. Despite this, the authors concluded, often very strongly, that 9INH was good, or was bad. These apparently contradictory findings resulted in greater confusion about benefits of, and indications for, treatment of LTBI. These may have contributed to the chronic underuse of LTBI treatment. Risk-benefit analyses of INH therapy in HIV infected individuals^49^,^50^ or other high risk groups^19^have estimated much greater, and consistent benefits. These analyses support current recommendations to test and treat LTBI only in individuals with high risk of reactivation to disease, if infected^13^,^14^.

**Table II:** Risk benefit studies of INH for tuberculin reactors

Study	Year	Age group (yr)	Preferred (INH or Not)	Margin of benefit* (days)

Low risk reactors				
Rose *et al*^44^	1986	10-80	INH	1- 16
Tsevat^45^	1988	20-80	No INH	4-17
Colice^46^	1990	30	INH	3-19
Jordan *et al*^47^	1991	20-35	INH	3-19
		50	No INH	2-33
Salpeter *et al*^48^	1997	35-70	INH	3-5
High risk reactors (HIV infected)				
Jordan *et al*^49^	1991	20-60	INH	285
Rose^50^	1998	Adults	INH	254

* Margin: means gain or loss of life expected with INH treatment, relative to no treatment. Superscript numerals denote reference numbers

There are many factors that increase the risk of re-activation. In view of this complexity, a web based algorithm has been developed which may provide some assistance in estimating that risk^51^. This algorithm, (available at *www.tstin3d.com*) incorporates information on age, TST or interferon gamma release assay (IGRA) results, country of birth, ethnic origin and clinical risk factors including chest radiographic findings. This information is entered and the algorithm calculates the likelihood of true latent infection, as well as the annual and cumulative life time risk of development of active TB.

One important consideration for any programme for screening and treatment for LTBI is its potential public health impact. This is of particular interest in LMIC given the resource implications of initiating such a programme. As seen in Table III, several studies have described the impact of LTBI screening and treatment programmes in many different settings^16^,^43^,^52^,^57^. In all these studies, the public health impact was very low. This is because patients did not participate in initial screening or failed to come for reading of tuberculin results, or did not report for medical evaluation of positive tests. In addition physicians were non-compliant with recommendations to prescribe treatment, and then patients refused to start LTBI therapy, or they were non-compliant if they did start. As a result, in all these studies fewer than 40 per cent of patients who could have benefited from LTBI therapy actually did so; in some studies as few as 10 per cent with presumed LTBI derived any benefit. If LTBI screening and treatment is being considered, then a great deal of attention must be paid to ensure that every stage of the programme is well organized, in order to enhance the public health benefit of this approach.

**Table III:** True impact of large scale screening programmes

Country	Total screened with TST (N)	TST positive (N)	Medically evaluated (N)	Started INH (N)	Completed INH		
					(N)	Of all positive TST (%)	Of all screened (%)

Canada^52^	19,001	4,292		814	452	11	2
USA^53^	4,840	2,039	1,528	853	716	35	15
Canada^54^	720	162	142	56	52	13	7
Canada^55^	33,146	7,668		2,600	1589	21	5
Canada^16^*	3,300	1,598	647	347	251	31	18
USA^56^	66,767	12,901		9,018	5746	37	9
Canada^45^	7,669	782	525	293	140	13	2
Uganda^57^	9,862	5227*	579	520	322	6	3

* In some studies the number of studies of TST positive is calculated based upon the expected number of TST positive in the original populations screened, because individuals did not accept TST or did not return for reading. Superscript numerals denote reference numbers

## Is testing for latent TB infection required before latent TB treatment?

A common clinical question is whether a TST or an IGRA is needed prior to giving LTBI therapy. This is particularly important in LMIC that are initiating LTBI therapy because tuberculin testing is not commonly performed. In some LMIC the tuberculin material itself (*i.e*., the PPD) is not available. In addition, WHO has recommended that LTBI therapy can be given without performing a TST^58^.

This question has been addressed in several randomized trials and in one systematic review. In these trials INH therapy was compared to placebo in HIV infected patients who had initial TST, but were randomized to active therapy or placebo regardless of TST results^59^–^64^. There is convincing evidence, (Table IV) that a positive TST predicts benefit from INH therapy. INH therapy is of minimal and non-significant benefit in HIV infected persons who are initially TST negative. Based on the systematic review of the earlier trials in HIV infected INH provides 60 per cent protection against disease in TST positive, but only 16 per cent benefit in TST negative persons^65^.

**Table IV:** Protection of INH against development of active TB in HIV infected individuals who were TST positive or TST Negative (from placebo controlled trials)

Studies	Year	Country	Risk reduction compared to placebo	
			Among TST positive persons % (range)	Among TST negative persons

Pape *et al*^59^	1993	Haiti	76 (0-94)	30 (0-85)
Whalen *et al*^60^	1997	Uganda	70 (32-87)	26 (0-70)
Hawken *et al*^61^	1997	Kenya	40 (0 - 77)	0 (0 - 45)
Mwinga *et al*^62^	1998	Zambia	72 (0-93)	18 (0 - 70)
Pooled^65^	2010	All	60 (35-76)	16 (0 - 40)
Samandari *et al*^38^	2009	Botswana**	90 (N/A)	14 (N/A)

* Pooled risk reduction from systematic review and meta-analysis of the above 4 studies plus Gordin *et al*^63^ and Garcia *et al*^64^

** The Botswana trial compared rate of reduction with 36 months INH vs. 6 months INH in HIV infected persons who were initially TST positive or TST negative, Hence the placebo control was given from month 7 to 36; both groups received INH for the first 6 months. Superscript numerals denote reference numbers

A trial just completed in Botswana confirmed these findings. In this trial HIV infected patients were randomized to 6 or 36 months of INH. In those who were initially TST positive, 36 months INH resulted in 92 per cent reduction in TB disease relative to those who received only 6 months INH. However, participants who were initially TST negative had only a 14 per cent reduction in TB disease with 36 months INH, compared to 6INH - a reduction that was not statistically significant^38^.

To date, no trials have addressed this question in IGRA positive and IGRA negative individuals. A recent systematic review found that the risk of disease in IGRA positive close contacts was similar to the rate of disease in close contacts who are TST positive^66^. IT was also found that the relative risk of disease in IGRA positive compared to IGRA negative was similar to the relative risk of disease in TST positive vs. TST negative. From this one can infer that benefit of INH will be found only in those who are initially IGRA positive. However, this extrapolation needs to be confirmed by evidence from studies specifically addressing this issue.

In summary, LTBI treatment is beneficial only in patients with a positive test for LTBI. It is important to remember that these studies were all conducted in settings with high TB incidence, and therefore presumed high levels of TB exposure and transmission. In addition, all study populations were HIV infected – and most had no access to anti-retroviral therapy. Hence we can assume they were very likely to have been immune suppressed. Therefore, these results should be considered highly relevant in low and middle income countries - TST negative persons should be given LTBI treatment only in exceptional circumstances. The one exception is in the situation of recent exposure. Following TB exposure and primary infection cell-mediated immunity will develop only after several weeks. In close contacts who are initially TST negative it is recommended that a second TST should be performed eight weeks after the end of exposure. By this time all tuberculin conversions would have occurred^67^. In individuals at very high risk of rapidly developing active disease, such as very young children (under 5), or HIV infected it is prudent to give INH while awaiting the results of the second TST.

## Conclusion: Why is there a debate about treating LTBI?

Despite clear evidence of benefits of LTBI therapy in persons with a positive TST and increased risk of re-activation, treatment of LTBI remains controversial. The reasons for the controversy may be the history of repeated cycles of enthusiastic recommendations of new regimens, followed by imprudent application of therapy, followed by disastrous adverse events (INH in 1971 and 2RZ in 2000). The controversy is further fuelled by studies documenting poor performance of programmes, leading many to conclude that the therapy is sub-optimal, rather than concluding that the programmes were suboptimal. Apparently contradictory risk-benefit studies, demonstrating marginal benefits in low risk persons, have added to the confusion.

As summarized (Table V) there are many parallels between treatment of LTBI and treatment of hypertension. Hypertension is also an asymptomatic condition with serious complications that can cause substantial morbidity and mortality, yet these can be successfully prevented with medication. In contrast to LTBI treatment, these medications must be taken for many years, and many are quite expensive. Treatment of hypertension is also associated with unpleasant side effects. Yet there is little or no debate about the value of treatment of hypertension.

**Table V:** Protection of INH against development of active TB in HIV infected individuals who were TST positive or TST Negative (from placebo controlled trials)

Hypertension	Latent TB infection

Asymptomatic condition Very serious complications – Death – Major disability Treatment is for years – Expensive medications – Potential serious side effects – Requires close monitoring and follow up BUT – no debate about Treating	Asymptomatic condition Very serious complications – Death – Major disability – AND transmission Treatment is max 9 months – Cheap medications – Potential serious side effects – Requires close monitoring and follow up WHY the debate about Treating??

Why then debate about treatment of LTBI?
